# A Novel Interpretation for Arterial Pulse Pressure Amplification in Health and Disease

**DOI:** 10.1155/2018/1364185

**Published:** 2018-01-16

**Authors:** Manuel R. Alfonso, Ricardo L. Armentano, Leandro J. Cymberknop, Arthur R. Ghigo, Franco M. Pessana, Walter E. Legnani

**Affiliations:** ^1^Facultad Regional Buenos Aires, Grupo de Investigación en Bioingeniería (GIBIO) and Escuela de Estudios Avanzados en Ciencias de la Ingeniería (EEACI), Universidad Tecnológica Nacional, Medrano 951, C1179AAQ Buenos Aires, Argentina; ^2^UMR 7190, Institut Jean Le Rond ∂'Alembert, CNRS and UPMC, Sorbonne Universités, 4 Place Jussieu, Boîte 162, 75005 Paris, France; ^3^Universidad Tecnológica Nacional, Medrano 951, C1179AAQ Buenos Aires, Argentina; ^4^Facultad Regional Buenos Aires, Centro de Procesamiento de Señales e Imagenes (CPSI) and Escuela de Estudios Avanzados en Ciencias de la Ingeniería (EEACI), Universidad Tecnológica Nacional, Medrano 951, C1179AAQ Buenos Aires, Argentina

## Abstract

Arterial pressure waves have been described in one dimension using several approaches, such as lumped (Windkessel) or distributed (using Navier-Stokes equations) models. An alternative approach consists of modeling blood pressure waves using a Korteweg-de Vries (KdV) equation and representing pressure waves as combinations of solitons. This model captures many key features of wave propagation in the systemic network and, in particular, pulse pressure amplification (PPA), which is a mechanical biomarker of cardiovascular risk. The main objective of this work is to compare the propagation dynamics described by a KdV equation in a human-like arterial tree using acquired pressure waves. Furthermore, we analyzed the ability of our model to reproduce induced elastic changes in PPA due to different pathological conditions. To this end, numerical simulations were performed using acquired central pressure signals from different subject groups (young, adults, and hypertensive) as input and then comparing the output of the model with measured radial artery pressure waveforms. Pathological conditions were modeled as changes in arterial elasticity (*E*). Numerical results showed that the model was able to propagate acquired pressure waveforms and to reproduce PPA variations as a consequence of elastic changes. Calculated elasticity for each group was in accordance with the existing literature.

## 1. Introduction

Pulse pressure amplification (PPA) is conventionally understood in clinical practice as the increase of pulse pressure (PP) amplitude as pressure waves propagate distally in the systemic network. Yet, PPA should rather be described as a distortion rather than an amplification of PP waves, represented by morphological alterations of pressure waveforms. Moreover, changes in PPA are associated with traditional cardiovascular risk factors, such as aging and hypertension [1, 2]. Indeed, a substantial decrease in mean diastolic pressure (perfusion) and a systolic central pressure increase (afterload) are observed in patients over 60 years old as a result of a progressive increase in arterial stiffness [3]. Consequently, a greater myocardial oxygen demand in the left ventricle and an impaired coronary perfusion are observed due to the decrease in mean arterial diastolic pressure [3]. Furthermore, in hypertensive patients, the decrease in large artery compliance (i.e., high values of arterial stiffness) is considered one of the major causes of PP increase. Additionally, hypertension is responsible for an increase in pulse wave velocity (PWV).

The study of PP propagation is therefore a major medical challenge and is essential to understand the dynamics of the circulatory system under normal or pathological conditions. Two different approaches have been used to efficiently describe the hemodynamics in the systemic network [4]. On the one hand, lumped parameter or 0D models [5–7] are particularly relevant when modeling interactions between the systemic network and other major systems (nervous, respiratory, and digestive) but are unable to describe pulse wave propagation. On the other hand, distributed 1D models [4] enable an efficient description of pulse wave propagation without the computational cost associated to 2D or 3D models.

In this work, we choose an alternative approach where long wave and perturbation theories allow us to derive a nonlinear dispersive and/or diffusive equation, like Korteweg-de Vries (KdV) equation, starting from the Navier-Stokes equations [8–11]. Behind this model is the idea that blood pressure (BP) waves can be considered as combinations of solitons. Laleg et al. [9] described this combination in details, through the nonlinear overlapping of two or three solitons. This model captures many of the phenomena observed in BP propagation, such as peaking (increase in amplitude), steepening (decrease in width), and changes in wave propagation velocity. Furthermore, McDonald found that an amplitude increase of arterial pulse is concomitant with a decrease in pulse width during the propagation of flow and pressure waveforms from the aorta to the saphenous artery in dogs [3], indicating a nonlinear rather than a linear behavior.

In previous works of our group [12, 13], a 1D arterial network was constructed in order to simulate the behavior of synthesized pulse pressure waveforms as a combination of solitons throughout the arterial tree. To this end, the pressure in each segment was computed using the KdV equation (KdVe), where vascular dimensions and elastic constants were obtained from the existing literature [14].

The main objective of this work is to use the arterial network previously described and the KdVe to compute the propagation of acquired pulse pressure waves through a human-like arterial tree and quantify the ability of our model to capture changes in PPA due to variations in arterial elasticity. To this end, numerical simulations will be performed, using a set of previously acquired central blood pressure (CBP) and peripheral blood pressure (PBP) waveforms from several individuals from four different groups: young, adult, hypertensive type I, and II.

## 2. Materials and Methods

In this section, we present the simple nonlinear model (KdVe) describing blood pulse pressure propagation in an artery. We then introduce a computational framework allowing us to use acquired CBP-PBP as inputs-outputs of our model. Next, we design a numerical experiment to assess the ability of our model to reproduce changes in PPA due to changes in vessel elasticity (*E*). Finally, using a set of CBP/PBP-acquired signals, we perform a global parameter estimation of the arterial elasticity (*E*) value for each patient and then perform a statistical analysis.

### 2.1. 1D KDV-Based Model Formulation

To explain BP waveforms and interpret the different phenomena that arise as they propagate along the arterial network, like the increase in amplitude and the decrease in width called “peaking” and “steepening” phenomena, respectively, this work introduces BP waves as a soliton combination. To understand the main behavior of soliton propagation, it is important to point out the following:

- Solitons have a bell shape and maintain their shape as they propagate.
- When solitons interact, they remain unchanged after the “collision,” except possibly for a phase shift.
- During interaction, the resulting shape is wider, and the amplitude is between the peak of the taller and the smaller one.
- Wave velocity and amplitude are dependent of *E*.
- Each soliton has its own velocity, because of this, the waves separate as they propagate to the periphery.

With the above attributes considered, it is then easy to explain phenomena like peaking, steepening, and PPA. Due to the different velocities (which depends on *E*), when the waves arrive at the periphery, the initial separation has changed. The different solitons are now more separated from each other creating a taller waveform. In young's (low *E*), this separation is bigger, adopting their respective original form: a taller and thinner one and a smaller and wider one, like the two typical bell shapes observed in the femoral artery.

In order to obtain the equations describing the dynamics of BP propagation along an elastic arterial segment, several authors [8, 11, 15, 16] propose:

(a) That large arteries are to be considered as elastic tubes and the fluid as incompressible
(b) For large arteries, the continuum approach for blood is valid and that viscosity can be neglected [8, 17, 18]. Following these authors, the evolution of pressure (*P*) in an arterial segment can be described as follows:
  (1) $$\begin{array}{c} P_{z}+d_{0}P_{t}+d_{1}PP_{t}+d_{2}P_{ttt}=0, \end{array}$$
  where *z* and *t* are the corresponding space and time variables and the subscripts of *z* and *t* indicate spatial and temporal derivatives. The equation coefficients are defined as follows:
  (2) $$\begin{array}{c} d_{0}=\frac{1}{c_{0}},\\ d_{1}=-\left(\alpha +\frac{1}{2}\right)\frac{1}{\rho {c_{0}}^{3}},\\ d_{2}=-\frac{\rho_{w}hR}{2\rho {c_{0}}^{3}}, \end{array}$$
  where the constant $c_{0}=\sqrt{Eh_{0}/2\rho R_{0}}$ determines the typical Moens-Korteweg velocity of a wave propagating in an elastic tube, when all nonlinear terms are neglected [19, 20], *E* is the elastic modulus (arterial stiffness), *h* is the wall thickness, *R* is the mean tube radius, *ρ* is the blood density, *ρ*_w_ is the wall density, and *α* is the moment flux correction coefficient.

### 2.2. Arterial System Model

**Figure 1:**
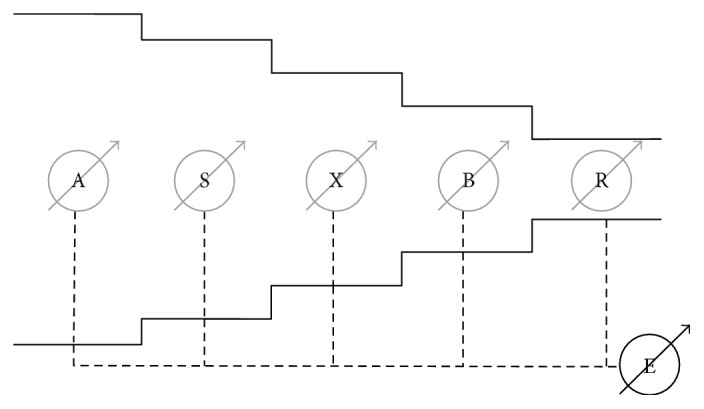
Diagram of the discrete compartmental model and the path used in this work. The path starts from the ascending aorta (A), continues through the subclavian (S), axillary, and brachial arteries (X and B), and ends in the radial artery (R).

In this work, a previously arterial network model was used [12]. This model consists of one long tapering *artery, composed of constant parameter vessels*, placed in a simple *cascading* order. In each of these segments, *the pulse pressure wave dynamics were modelled by* (1) *describing only forward soliton interactions*. At the inlet of the network (aorta), an acquired CBP is imposed. The computed PBP at the outlet of the final segment constitutes the output of the model (Figure 1).

The arterial network starts from the ascending aorta (A), continuing through the subclavian (S), axillary (X), and brachial arteries (B), and finally ending in the radial artery (R) (Figure 1).

The length, radius, thickness, and elastic values used to describe each segment are shown in Table 1. The wall density (*ρ*_w_) and fluid density (*ρ*) are 1.06 g/cm^3^ and 1.05 g/cm^3^, respectively. The moment-flux correction coefficient *α* is set as 1 *in accordance with the inviscid assumption and with experimental findings*.

As the KdVe is a stiff equation, classical numerical methods are numerically unstable unless an extremely small step size is used [21]. We therefore chose a spectral numerical scheme to perform the numerical integration of the KdVe, as recommended in [22, 23]. A 4th order exponential time differencing Runge-Kutta (etd4rk), developed by Cox and Matthews [24], was selected, and its efficiency was previously evaluated by our group [25].

### 2.3. Clinical Measurements

Radial artery BP was acquired using the tonometry technique (Millar Inc., Houston, Texas, USA), in a baseline state at a supine position, and calibrated using sphygmomanometric measurements. CBP was determined by means of a transfer function using a previously validated algorithm (SphygmoCor, Atcor Medical, Illinois, USA). Obtained BP waveforms were separated into four groups:

- “Young group” aged 20 to 29 years (*n* = 15)
- “Adult group” aged 40 to 69 years (*n* = 13) with normal BP
- “Hypertension type I (HTI) group” aged 40 to 69 years (*n* = 15)
- “Hypertension type II (HTII) group” aged 40 to 69 years (*n* = 13)

HTII group was composed of fully developed hypertensive patients while HTI subjects were only in an initial stage of hypertension.

It is worth mentioning that for a person in a supine position, diastolic and mean pressures are considered constant throughout the arterial system [3].

### 2.4. Modeling in Health or Disease

It is well known that, in a disease condition, systolic PBP changes due to vascular changes in the *E*, *h*, and *R* values. Nevertheless, we simplified the analysis and focused only on the influence of changes in *E*. To verify the validity of this hypothesis, a typical acquired CBP waveform was introduced as the initial condition, and the elastic values of the cascade model were increased and decreased by 25% (*for further details, see* Results). Later, using the acquired CBP wave of each subject, we reproduced the measured PPA using the global parameter estimation strategy described below.

### 2.5. The Global Fitting Procedure

Two different parameter estimation strategies can be used to evaluate the parameters of an arterial network. A local approach would search for the best set of parameters in each segment independently of the other segments. Such a strategy would require an acquired wave at the end of each segment. On the other hand, a global approach modifies the parameters of each tube by the same percentage. Therefore, only a couple of acquired signals is necessary.

In this work, we chose the latter approach and used a single global parameter to uniformly modify the *E* value in each segment of the arterial tree model.

Because of the nature of the pressure wave, the global parameter estimation strategy can be of two types: *morphological*, where the coefficients (only *E* in this case) are modified to obtain a wave whose morphology properly fits the shape of the acquired signal; or *parametric*, where a set of parameters is calculated from the acquired signal and the model output is modified to match those parameters. We decided to use a global parametric estimation approach, with the systolic peak pressure as the reference parameter and a tolerance less than 1 mmHg for the error between the acquired and computed systolic peak pressure.

**Figure 2:**
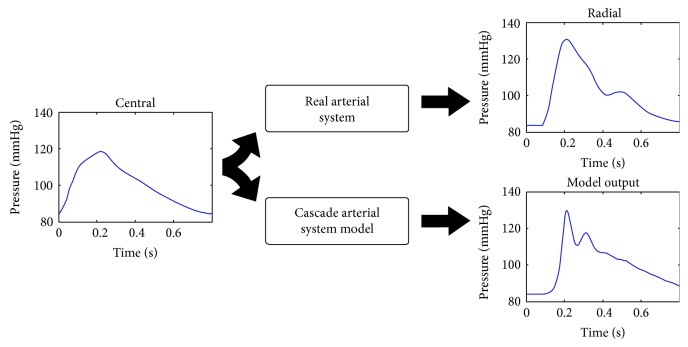
Procedure performed to validate the output of the model.

The fitting procedure was based on an algorithm similar to the bisection search, knowing that increasing *E* will decrease the systolic peak and vice versa. Physiological lower and upper limits were imposed for *E* variations, and a maximum iteration counter for nonconverging cases was added. Finally, different indices, like goodness of fit (GOF) in time and frequency, cross-correlation (XCOR), cross-coherence (COH), and cross-phase coherence were calculated in order to quantify the similarity between computed and acquired pressure signals (Figure 2).

#### 2.5.1. Goodness of Fit

Goodness of fit (GOF) is a measure of the discrepancy between the observed *x* and the expected xref values. It is calculated as follows:

(3) $$\begin{array}{c} GOF\left(i\right)=1-{\left\|\frac{xref\left(\text{:},i\right)-x\left(\text{:},i\right)}{xref\left(\text{:},i\right)-mean\left(xref\left(\text{:},i\right)\right)}\right\|}^{2}. \end{array}$$

#### 2.5.2. Cross-Correlation

Cross-correlation is a measure of the similarity between two time series, *f* and *g*, as a function of a time lag. It is calculated as follows:

(4) $$\begin{array}{c} \left(f⋆g\right)\left(\tau \right)≝\int_{-\infty }^{\infty }f^{*}\left(t\right)g\left(t+\tau \right)dt, \end{array}$$

where *f*^∗^ denotes the complex conjugate of *f* and *τ* is the time lag.

#### 2.5.3. Coherence

Coherence indicates how well *x* corresponds to *y* for each frequency. The magnitude-squared coherence is a function of the power spectral densities, *P*_*xx*_(*f*) and *P*_*yy*_(*f*), of *x* and *y*, and the cross-power spectral density, P_*xy*_(*f*), of *x* and *y*. It is calculated as follows:

(5) $$\begin{array}{c} C_{xy}\left(f\right)=\frac{{\left|P_{xy}\left(f\right)\right|}^{2}}{P_{xx}\left(f\right)P_{yy}\left(f\right)}. \end{array}$$

### 2.6. Statistical Analysis

The results were obtained from simulations. Data were expressed as mean ± standard deviation (SD). A statistical analysis was conducted with Mann–Whitney and Kruskal-Wallis ANOVA tests. A value of *p* < 0.05 was considered to be statistically significant.

The Kruskal-Wallis *H* test is a nonparametric test which is used instead of a one-way ANOVA. It is essentially an extension of the Wilcoxon rank-sum test to more than two independent samples. The Kruskal-Wallis test becomes quite useful in particular when group samples strongly deviate from normal (generally for small sample sizes) and group variances are quite different. Unlike ANOVA, Kruskal-Wallis makes no assumption about distribution.

The statistical analysis was performed using SPSS version 23 (SPSS Inc., Chicago, Illinois, USA) and Matlab® version 2014.

## 3. Results

**Figure 3:**
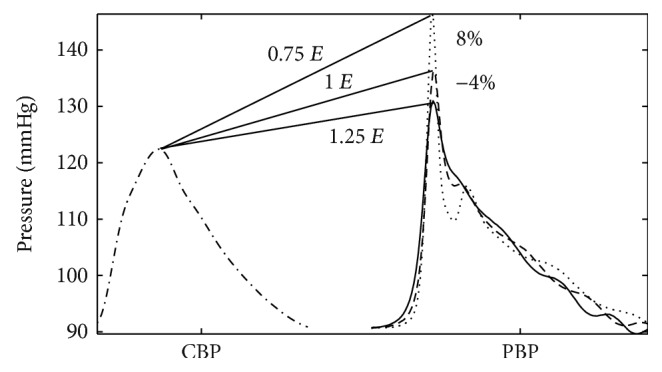
Changes in blood pressure (BP) as a result of the variations in *E* for an individual of the adult group. Central BP (-.-), radial BP with initial *E* (- - -), radial BP with *E* decreased by 25% (…), and radial BP with *E* increased by 25% (__).

Figure 3 shows the output of the model for different *E* values. As it can be seen, PPA is affected by changes of *E* as it increases and decreases by 25%. Furthermore, CBP wave morphology is affected as it propagates towards the periphery for both peaking and steepening phenomena.

**Figure 4:**
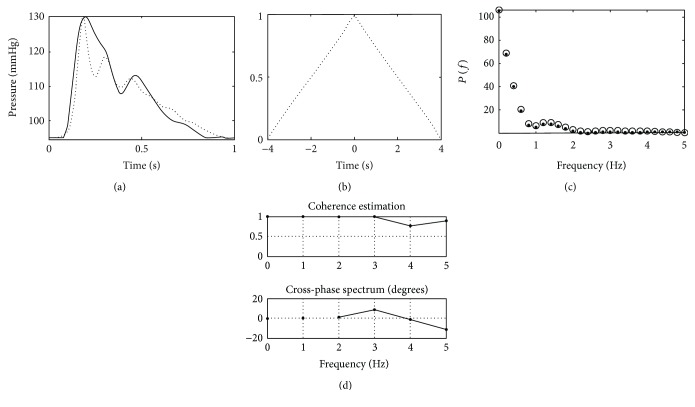
(a) Measured (solid) and obtained (dotted) radial artery pressure waveform. (b) Cross-correlation between measured and obtained PBP. (c) Frequency response of measured (o) and obtained (.) PBP waveforms. (d) Amplitude and phase of coherence between measured and obtained PBP waveforms.

Figure 4(a) shows the measured and obtained PBP in a typically healthy adult. We observe that the model is able to describe the peak of the measured PBP and morphology of the waves. This is quantified with a GOF of 0.9. Cross-correlation between measured and computed signals is shown in Figure 4(b). The approximated triangular shape can be understood as a high degree of similarity. The frequency response of both measured and computed PBPs is shown in Figure 4(c), where almost no harmonic alterations were made by the model. The GOF of the frequency response is almost 1. Finally, coherence is shown in Figure 4(d) and we observe that, for frequencies smaller than 2 HZ, the acquired and computed module and phase shifts are equal. For the rest of the sample, very close results were obtained.

**Figure 5:**
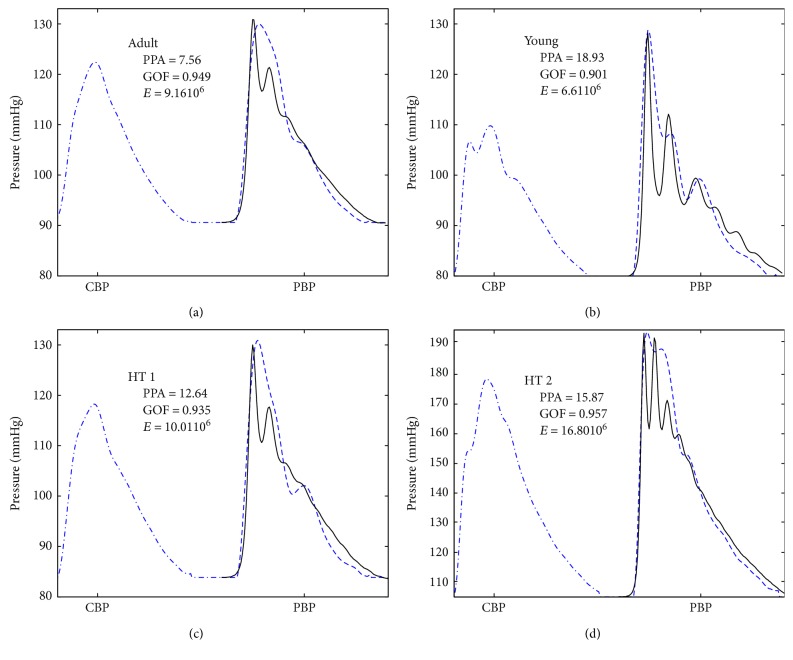
Typical case for (a) adult, (b) young, (c) hypertensive type 1, and (d) hypertensive type 2. Central BP waveform (-·-). Radial BP measured for each subject (- -). Result of the application of the model with adjusted *E* (___).

Figure 5 shows the acquired CBP and PBP and the model output for a typical subject of each group.  Figure 5(a) shows a typical waveform of the adult group, with a PPA of 7.56 mmHg and a calculated *E* of 9.16 × 10^6^ Dyn/cm^2^. The similarity between measured and model outputs was quantified by the GOF of 0.949. In Figure 5(b), a typical waveform from the young group is shown. We observe that, as expected, the PPA is significantly *higher* than in Figure 5(a) and that the elastic value is a bit *lower*. In Figures 5(c) and 5(d), waveforms from the HTI and HTII groups are displayed. Waveforms in Figure 5(c) are similar to those of the adult group presented in Figure 5(a), even though higher PPA and *E* values were computed. These differences are accentuated in Figure 5(d).

In order to analyze this trend by population, calculated parameters are shown in Table 2 and expressed as mean ± SD. Excluding the young group, age and body mass index (BMI) were similar for the other groups. Heart rate (HR), weight, and height were evenly distributed between the four groups. Systolic central blood pressure (SCBP) and PP were significantly lower than radial SBP and PP within all four groups. SBP and diastolic blood pressure (DBP) for central and peripheral sites increased gradually from the young group to HTII. PP values remain almost identical for the first three groups, with a significant change for the HTII group (*p* < 0.05).

A gradual increase is observed in the computed elastic value (*E*) from its lowest value in the young group to the HTII group, showing that the arterial elastic properties of young and hypertensive type II patients are, respectively, lower by 20% and higher by 40% with respect to those of a healthy adult. Statistically significant differences were observed in the systolic pressure values (CBP and PBP) and were in accordance with group classification. Finally, the GOF was above 80% for the young and above 90% for all other groups. Furthermore, analysis of the PPA and *E* values for women only showed that PPA results were almost the same (10 mmHg) than those of the adults, HTI, and HTII. Moreover, *E* values for the HTI were also almost equal to those of the adults.

## 4. Discussion

In this paper, we take a different approach than the traditional distributed 1D modeling of arterial segments. Based on the hypothesis that soliton interactions can describe arterial pressure waveforms [11], we used the KdVe to model pressure wave dynamics in an arterial network, with the useful effect of a reduced computational cost. To this end, we used the cascade arterial tree model proposed in a previous work by our group [12]. We first validated the model using acquired data (radial artery and central). Secondly, we tested the ability of the model to describe PPA dependence on *E* variations. Therefore, in all the experiments, only the dependence on *E* was accounted for, leaving more complicated multiple-parameter estimation for a further study.

To the best of our knowledge, a KdVe model capable of describing alterations of the arterial wall properties has not yet been reported. The main objective of this original work was accomplished. The propagation of acquired pulse pressure waves through a human-like arterial tree was quantified showing the ability of our model to capture changes in PPA due to variations in arterial elasticity associated to different pathological conditions. The model was able to reproduce the main features of the PP propagation, including the singular PPA phenomenon: when *E* increases, there is a decrease in PPA and vice versa (see Figure 3).

All the tests selected to quantify the similarities between the acquired and computed pressures provided substantial support for the results of the oversimplified 1D model for the propagation of waves in a complex network. For instance, the goodness of fit provided by the NMSE is between 80 and 90%, which constitutes an acceptable waveform representation for the developed model (see Table 2, last column).

*It is noteworthy that, unlike the traditional approach, where the effects of PPA are described through the wave reflection phenomenon* [3]*, this study analyzes the evolution of nonlinear waves traveling from the aortic arch, whose interaction determines the morphology of the peripheral wave*. The morphological dependence on *E* can be easily described by means of the soliton theory, where waves with different amplitudes travel at different speeds, due to an amplitude-velocity relationship. In this sense, at low speeds (i.e., low *E*, as found in young individuals with no vascular disease), the different solitary waves that shape CBP are more separated when they reach the periphery, where peaking and steepening phenomena are observed. Increased *E*, caused by aging or hypertension, diminishes this separation and consequently a smaller PPA is observed. In fact, our study showed that PPA decreases and *E* increases as a result of aging or hypertension, which is in accordance with previous results (see Table 2) [26, 27].

By applying the Moens-Korteweg formula, the mean pulse wave velocity was estimated as 878, 960, 1000, and 1141 cm/s for the young, adult, HTI, and HTII groups, respectively. For the adult group, the PWV value is close to reference values for the same population but in the carotid-femoral arteries [28, 29]. In the HTII group, the equivalent *E* modulus is 41% higher than in the adult group, which implies an increase of 19% in PWV, close to the 18.75% increase reported in [28]. This stiffness is linked to a 53% increase in PPA (9.3 to 14.3 mmHg), which is close to the increase of 48% between normal and HTAII [28, 29].

The young group showed lower stiffness compared to the adult group; in this case, the equivalent *E* modulus fell by 18% to allow for a proper fit (see Figure 5). This decrease in *E* represents a 9% decrease in PWV consistent with the literature [28], concomitantly to a 113% increase in PPA, in accordance with other works [1] in which the same trend has been found.

Typical trends among the four groups, youth, adults, HTI, and HTII, are presented in Figure 5 where the model fits the input signals, reproducing the PPA with a calculated *E* module. These typical cases faithfully represent the distinctive characteristics reported in the literature.

## 5. Study Limitations

In the present study, CBP and PBP waves were acquired noninvasively. Radial artery BP waves were obtained using the tonometry technique, and CBP was determined by means of a transfer function using a previously validated algorithm (SphygmoCor, Atcor Medical, Illinois, USA).

A simple 1D KDV-based model was used for wave propagation. The advantage is a good approximation in amplitude change and stiffness assessment, in addition to the reduced computational cost in relation to typical 1D models. However, wave morphology could be improved. To this end, the model could be extended with blood or wall viscosity and/or wall viscoelasticity. Moreover, it could be improved with a continuous tapering geometry for a more relevant adaptation of the prevailing geometry of the arterial system.

Arterial stiffness was used as estimation parameter to assess PBP in a simple way. To this purpose, wall thickness and vessel radius could also be used in a more complex procedure.

Calculation of other hemodynamic parameter or morphology-dependent risk factor should be addressed carefully. However, Figure 4 shows that wave dynamics are well characterized and that the model reproduced the peak location (used for example for augmentation index). Studies like [30], where the relation of HR, PPA, and stiffness is assessed, could be made with appropriate data.

## 6. Conclusion

In this work, a study of a nonlinear model for arterial pulse pressure propagation was carried out under different conditions on the *E* values. The ability to use acquired data as input for our model was verified and good agreement was found between measured and computational results. Moreover, the representation of PPA variations as a consequence of changes in *E* was also verified. The *E* value was adjusted to recreate morphological changes, and the resulting PPA variations were in accordance with previous studies. As a result, this model is capable of simulating aging and hypertension and can be useful to explain the clinical implication of PPA.

With this model, and using a SphygmoCor measurement, arterial stiffness could be assessed without using an ultrasound system for measuring arterial diameter and without considering the indirect measurement of the PWV.

In clinical practice, only concepts of vascular impedance and pulse wave velocity are widely used to assist clinical diagnosis and treatment, and few integrated 0D models comprising the complete description of the heart and vessels have seen use in clinical practice. Currently, some 1D models have been successfully applied in the context of clinical diagnosis of pathological changes in the cardiovascular system (such as hypertension and atherosclerosis). The proposed KdV model has proven to be a good clinical approach to assess the hypertensive state with or without treatment, and the results are in accordance with the literature.

## Figures and Tables

**Table 1:** Arterial segments and their coefficients used for simulations, taken from [14].

Segment	Length (*L*, cm)	Radius (*R*, cm)	Wall thickness (*h*, cm)	Elasticity (*E*, 10^6^ Dyn/cm^2^)
Ascending aorta	4.000	1.450	0.163	4
Aortic arch	2.000	1120	0.132	4
Subclavian	3.400	0.420	0.067	4
Axillary	6.100	0.360	0.062	4
Axillary	5.600	0.310	0.057	4
Brachial	6.300	0.280	0.055	4
Brachial	6.300	0.260	0.053	4
Brachial	6.300	0.250	0.052	4
Brachial	4.600	0.240	0.050	4
Radial	11.700	0.160	0.043	8
Radial	11.700	0.160	0.043	8

**Table 2:** Clinical parameters.

	Young	Adults	HTI	HTII
Age (years)	26 ± 1	53 ± 9^a^	56 ± 10^a^	57 ± 11^a^
Sex	8F/7M	11F/2M	9F/6M	8F/5M
HR (bpm)	67 ± 9	72 ± 11	72 ± 10	71 ± 14
Weight (kg)	62 ± 9	74 ± 14	79 ± 7	77 ± 17
Height (cm)	171 ± 9	163 ± 9	166 ± 7	166 ± 10
BMI (kg/cm^2^)	21 ± 2	28 ± 5^a^	29 ± 4^a^	28 ± 4^a^
SCBP (mmHg)	104 ± 8	123 ± 10^a,b^	119 ± 9^a,b^	153 ± 12^a^
DCBP (mmHg)	70 ± 8	87 ± 5^a^	80 ± 9^a^	93 ± 17^a^
PPCBP (mmHg)	34 ± 6^b^	36 ± 10^b^	39 ± 9 ^b^	61 ± 20
SPBP (mmHg)	124 ± 11^b^	133 ± 9^b^	131 ± 11^b^	168 ± 13
PPPBP (mmHg)	54 ± 11^b^	46 ± 10^b^	51 ± 11^b^	75 ± 22
PPA (mmHg)	19.90 ± 5.37	9.33 ± 2.34^a^	11.47 ± 4.82^a^	14.30 ± 9.44
*E* (10^6^ Dyn/cm^2^)	8.04 ± 1.37	9.81 ± 1.97^b^	10.68 ± 1.85^a,b^	13.80 ± 2.70^a^
GOF	0.86 ± 0.06	0.90 ± 0.09	0.91 ± 0.04	0.90 ± 0.06

^a^ *p* < 0.01 with respect to the young group. ^b^*p* < 0.01 with respect to the HTII group; HR: heart rate; BMI: body mass index; PP: pulse pressure: CBP: central blood pressure; PBP: peripheral blood pressure; SCBP and SPBP: systolic central and peripheral blood pressure, respectively; DCBP: diastolic central blood pressure; PPA: pulse pressure amplification; *E*: elastic value; GOF: goodness of fit.
